# Strong Optical Coupling of Lattice Resonances in a Top-down Fabricated Hybrid Metal–Dielectric Al/Si/Ge Metasurface

**DOI:** 10.1021/acs.nanolett.3c05050

**Published:** 2024-03-01

**Authors:** Paul Oleynik, Fritz Berkmann, Sebastian Reiter, Jon Schlipf, Markus Ratzke, Yuji Yamamoto, Inga Anita Fischer

**Affiliations:** † Experimentalphysik und Funktionale Materialien, Brandenburgische Technische Universität Cottbus-Senftenberg, Erich-Weinert-Straße 1, 03046, Cottbus, Germany; ‡ Department of Physics, Sapienza University of Rome, 00185 Rome, Italy; § IHP−Leibniz Institut für Innovative Mikroelektronik, Im Technologiepark 25, 15236, Frankfurt (Oder), Germany

**Keywords:** metamaterials, semiconductors, hybridization, optoelectronics

## Abstract

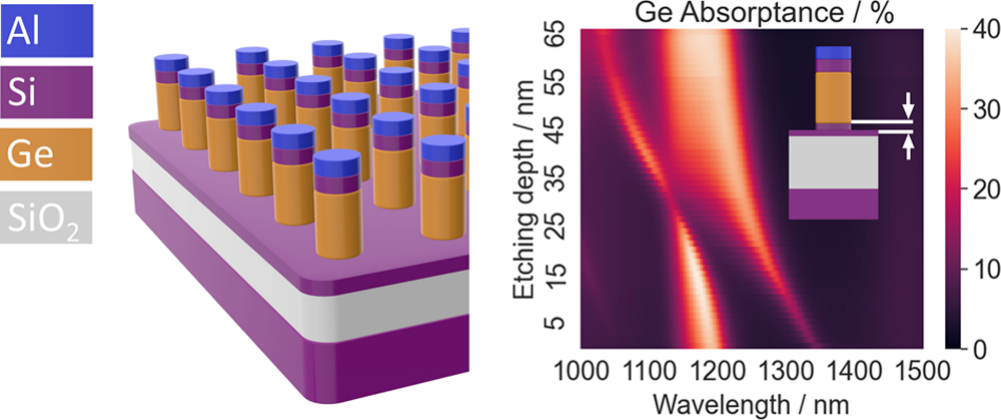

Optical metasurfaces enable the manipulation of the light–matter interaction in ultrathin layers. Compared with their metal or dielectric counterparts, hybrid metasurfaces resulting from the combination of dielectric and metallic nanostructures can offer increased possibilities for interactions between modes present in the system. Here, we investigate the interaction between lattice resonances in a hybrid metal–dielectric metasurface obtained from a single-step nanofabrication process. Finite-difference time domain simulations show the avoided crossing of the modes appearing in the wavelength-dependent absorptance inside the Ge upon variations in a selected geometry parameter as evidence for strong optical coupling. We find good agreement between the measured and simulated absorptance and reflectance spectra. Our metasurface design can be easily incorporated into a top-down optoelectronic device fabrication process with possible applications ranging from on-chip spectroscopy to sensing.

Optical metasurfaces consisting of two-dimensional arrangements of subwavelength-sized metallic or dielectric nanostructures have attracted strong interest for their ability to enable the manipulation of the light–matter interaction in ultrathin layers, with applications ranging from ultrathin lenses or filters to sensing.^1−5^ Metallic nanoparticles supporting plasmonic resonances as building blocks for such metasurfaces exhibit strong field confinement in their vicinity, while resonances are typically broad. Dielectric nanoparticles, on the other hand, support narrow resonances but exhibit a lower field confinement. Hybrid metal dielectric metasurfaces have attracted growing attention in recent years, motivated by the desire to combine the advantages of dielectric and metallic nanoresonators by utilizing the interaction of plasmonic and photonic modes.^6,7^ In particular, the regime of strong optical coupling between modes in hybrid systems, which can be attained when the coupling strength exceeds the damping rates of the coupled modes, yields resonances with properties of both individual modes, and the effects of strong optical coupling in hybrid metal dielectric systems have been investigated, e.g., to enable the excitation of dark plasmons^8^ or for application in higher harmonic generation.^9^ Proposed hybrid metal dielectric systems comprise the combination of plasmonic nanostructures with extended dielectric structures such as waveguides^10^ or microresonator cavities^11^ as well as the combination of dielectric nanostructures with extended metallic structures.^12,13^ Hybrid plasmonic–photonic metasurfaces, in which both the metallic and the dielectric constituents are nanostructured, include core–shell^14,15^ as well as stacked^16,17^ configurations. In this context, vertical stacks of metallic and dielectric nanostructures that result from one single top-down structuring step are particularly advantageous for their ease of fabrication, and such hybrid structures have been investigated, e.g., for applications in refractive index sensing.^17^

Here, we investigate a hybrid metal–dielectric metasurface consisting of a sandwich of disk-shaped Al antennas with Ge nanocylinders separated by a thin Si disk and arranged in a square lattice on top of a silicon-on-insulator (SOI) substrate. Our metasurface is based on Complementary-Metal-Oxide-Semiconductor (CMOS)-compatible materials. It is designed for straightforward incorporation into an optical device, such as a PIN photodetector, and can be fabricated using one single lithography and etching step. Using FDTD simulations, we investigate the influence of changes in geometry parameters on absorptance spectra and interpret the results in the context of strong optical coupling between collective lattice resonances in our hybrid metasurface. Our results are supported by good agreement between simulated and measured absorptance and reflectance spectra. Given the choice of materials as well as its straightforward fabrication, our hybrid metasurface design can potentially be integrated into a Si-based foundry process. This can serve to make its optical properties accessible, e.g., to applications in wavelength-selective photodetection for on-chip spectrometers, in a scalable, cost-effective approach.

A schematic image of the metasurface is shown in Figure 1a. The commercial solver Ansys Lumerical was used to carry out all FDTD simulations, using the broadband fixed angle source technique (BFAST,^18^) to realize nonperpendicular incidence of light. The perfectly matched layer (PML) boundary conditions (type “stretched coordinate PML”) above and below the array were realized using the profile “Steep angle” with 64 layers. The Yee-Cells had a maximum mesh step of 10 nm in every direction, and the simulation time was limited by an auto shutoff of 10^–5^. We used literature values for the optical properties of Al,^19^ Si,^20^ and SiO_2_^19^ and measured values for those of Ge. The complex refractive indices of Al, Si, and Ge for the wavelength range 1000 ≤ λ ≤ 1500 nm investigated here are shown in the Supporting Information (Figure S1).

**Figure 1:**
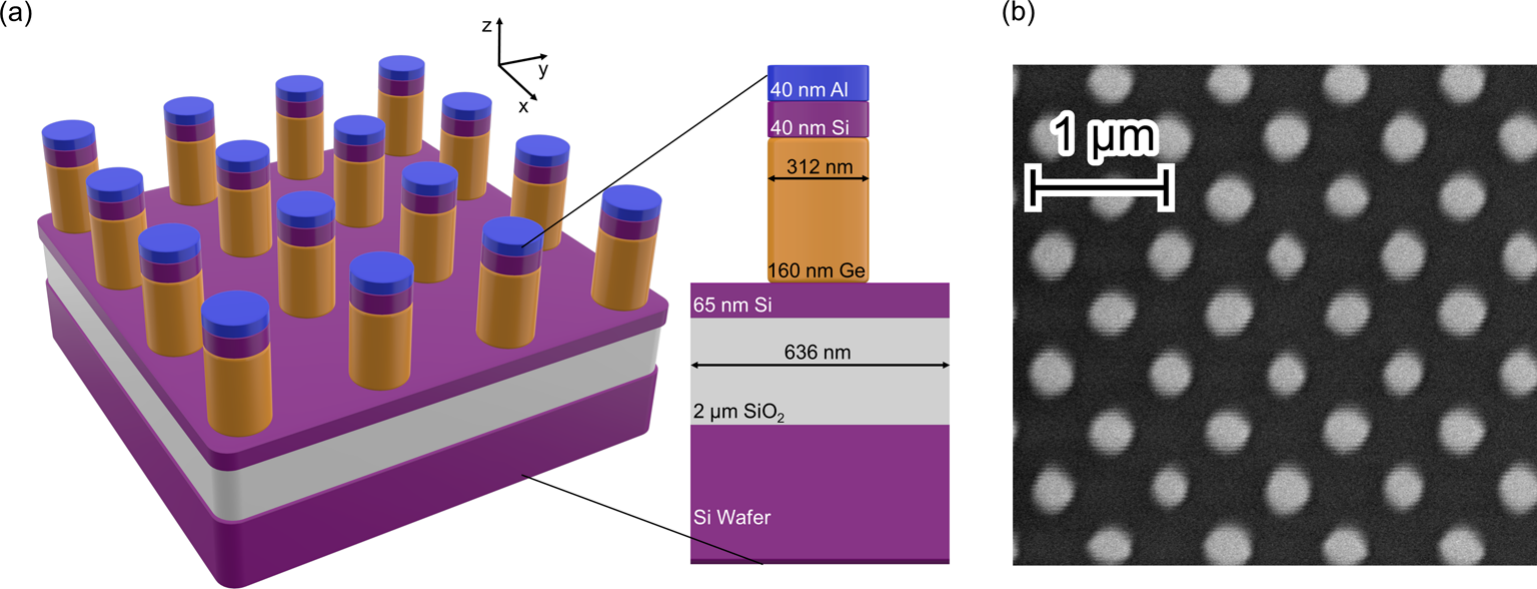
(a) Schematic image and cross-sectional view of the hybrid metasurface square lattice and layer structure. (b) Scanning electron microscopy image of the fabricated metasurface (top view). As a result of interference lithography, the diameters of the individual nanopillars show variations.

The metasurface fabrication process started from a SOI substrate with a thinned-back Si layer, on which 160 nm of Ge were deposited by chemical vapor deposition (CVD). This epitaxial growth was followed by a cyclic annealing step with the aim of reducing the threading dislocation density and improving layer quality.^21^ A cap layer consisting of 40 nm of Si was then deposited by CVD, followed by a second annealing step at 800 °C to improve the layer quality. Layer thicknesses were measured by reflectometry. Finally, an Al layer with a thickness of ∼40 nm was deposited via e-beam evaporation. The structuring of the metasurface as a square 3 × 3 mm^2^ array was carried out via optical lithography, using the I-line at 365 nm of a mercury arc lamp, followed by a single dry-etching step. In order to attain the small lateral structural sizes, a positive resist (AZ MIR 701, diluted 4:1 in ethyl lactate) with a low thickness of ∼300 nm was used. Furthermore, the structures were fabricated by interference lithography, i.e., while a square lattice of disks with a lattice pitch of 900 nm was defined on the photolithography mask, interference effects during the lithography led to the formation of additional resist structures positioned in the center of the lattice unit cells defined by the lithographic mask, resulting in a lattice pitch of (900/√2) ≈ 636 nm for the fabricated array (Figure 1b). This allowed us to go significantly below the expected spatial resolution of our optical lithography setup but introduced variations in lateral structural sizes. Additionally, this interference lithography not only limits us to square arrays but also restricts the attainable lattice pitch, precluding us from investigating a range of lattice pitches. Finally, the Al and the semiconductor layers were etched in a single hydrogen bromide (HBr) dry etching step in an inductively coupled plasma reactive ion etching (ICP-RIE) system. Atomic force microscopy (AFM) measurements of the etched structures using a SMENA (NT-MDT LLC) in contact mode revealed a total pillar height of 305 nm, indicating that the final etching step had also etched the bottom Si layer of the structure.

Spectroscopic measurements of the reflectance spectra were carried out using a LAMBDA 1050 UV/vis/NIR (ultraviolet-visible-near-infrared) spectrometer (PerkinElmer Inc.) with a “Total Absolute Measurement System” (TAMS) and an InGaAs detector. We used a rectangular spot of 1 × 2 mm^2^ to obtain spectra at different excitation angles. We illuminated our sample with p-polarized and s-polarized light at various incident angles (10°, 20°, and 30°) and measured the reflectance spectra in the range of 1000–1500 nm. Furthermore, we extracted the absorptance based on transmittance measurements performed under perpendicular incidence in the LAMBDA 1050 spectrometer in combination with reflectance measurements that were carried out using a HYPERION II FT-IR (Bruker Corp.) with an InGaAs detector on a 20 × 20 μm^2^ square area. We note that the presence of a microscope objective in the FT-IR setup leads to a distribution of incidence angles for the incident light. The absorptance was obtained using a deembedding approach that takes into account multiple reflections in the underlying substrate layers.^22^

Our metasurface design (Figure 1a) is intended for facile integration into optoelectronic devices, as the presence of the continuous Si layer ensures that individual elements of the metasurface can be contacted electrically. One possible application is in wavelength-sensitive photodetectors for on-chip spectrometers. A full device integration requires the realization of a top electrode that also connects all metasurface elements, necessitating additional fabrication steps. In this work, our goal is to investigate the optical properties of the hybrid metasurface without a top electrode as a first step. As such, we focus on simulation results for the wavelength-dependent absorptance in the semiconductor layers as a function of geometry parameters; this can straightforwardly be related to photocurrents in devices based on this metasurface.^23^ We restrict our investigation to a wavelength range of 1000 nm ≤ λ ≤ 1500 nm, which approximately covers the range of photon energies that is limited by the bandgaps of Si and Ge. In this wavelength range, Si is transparent, and only absorption in the Ge has to be considered.

The optical behavior of our hybrid metasurface can, in principle, be influenced by the interplay of multipole Mie resonances in the individual Ge nanocylinders as well as collective lattice resonances resulting from coupling of the nanocylinders in the square array.^24,25^ Similarly, both localized multipole Mie resonances in the Al nanodisks as well as extended surface plasmon resonances appearing in the Al nanodisk lattice can influence optical properties.^26^ A comparison of simulated absorptance spectra of a single unit cell and the structure with periodic boundary conditions shows that the optical properties of the hybrid metasurface are strongly influenced by interparticle coupling effects: The absorptance in the array differs considerably from the wavelength-dependent absorptance calculated for the Ge disk of a single structure and exhibits two peaks in the wavelength range examined here (Figure 2a).

**Figure 2:**
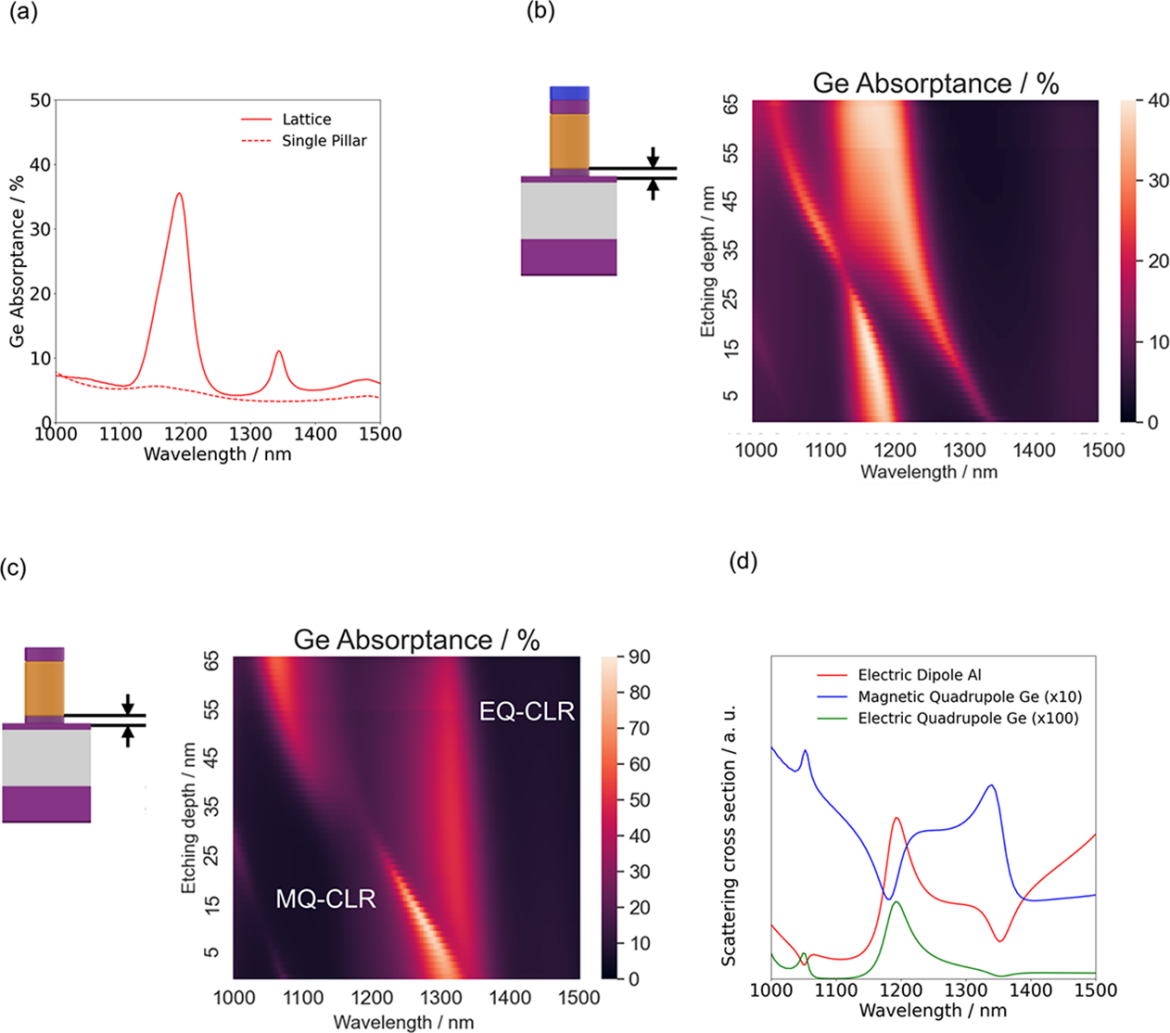
(a) Simulated absorptance spectra in the Ge layer for a single pillar (dashed line) in comparison to the nanopillar lattice (solid line) show that collective lattice resonances can be expected to strongly influence optical properties. (b) Absorptance spectra of the hybrid metasurface as a function of etching depth in the bottom Si layer, where the magnitude of the absorptance is indicated by color, show signatures of avoided crossing of the peaks as the etching depth is varied. (c) Avoided crossing behavior is absent in the absorptance peaks obtained for a purely dielectric metasurface upon variation of the etching depth. (d) A multipole analysis indicates the presence of electric dipole resonances in the Al as well as electric and magnetic quadrupole resonances in the Ge for an etching depth of 0 nm. The scattering cross sections corresponding to the magnetic and electric quadrupole resonances in the Ge have been multiplied by factors of 10 and 100, respectively, to increase their visibility.

Geometry parameters can be expected to influence the spectral positions and shapes of the absorptance peaks. A variation of the etching depth of the bottom Si layer has the most striking influence on the absorptance peaks, which show avoided crossing behavior as the etching depth is increased to its maximum value given by the thickness of the bottom Si layer (Figure 2b). We extract a normal mode splitting of 80 meV from a coupled, lossless oscillator model fit to the data.^27^ Details of the model and the fit are given in the Supporting Information (Figure S2). This avoided crossing behavior is notably absent in the spectra simulated for a purely dielectric metasurface, in which the Al caps are omitted, while all other geometry parameters are kept identical (Figure 2c).

A multipole analysis indicates that the optical properties of the hybrid metasurface in the wavelength range investigated here are dominated by electric quadrupole (EQ) and magnetic quadrupole (MQ) collective lattice resonances (CLR) in Ge and electric dipole collective lattice resonances (ED-CLR) in Al (Figure 2d). We used the electric fields computed by the FDTD simulation software for a unit cell of the hybrid metasurface, comprising both the Al and the semiconductor disks, to perform the multipolar decomposition. The analysis was carried out using the open-source package MENP (Multipole Expansion for Nanophotonics),^28^ which is based on the exact multipole expansion method proposed in ref (29). It is known that multipolar decompositions depend on the choice of origin of the coordinate system; here, we chose the center of the Al and Ge disks for all geometries to facilitate comparison. Plots of the electric and magnetic fields at resonance wavelengths for structures with different etching depths also show clear signs of EQ and MQ resonances in the semiconductor layers of the hybrid metasurface (Figure 3). Plots of the fields in the all-dielectric metasurface are shown in the Supporting Information, where the influence of other geometry parameters (thicknesses of the top Si disk, the Ge disk, and the Al disk as vertical geometry parameters as well as disk diameter and lattice pitch as lateral geometry parameters) on the spectral positions and shapes of the absorptance peaks is also discussed.

**Figure 3:**
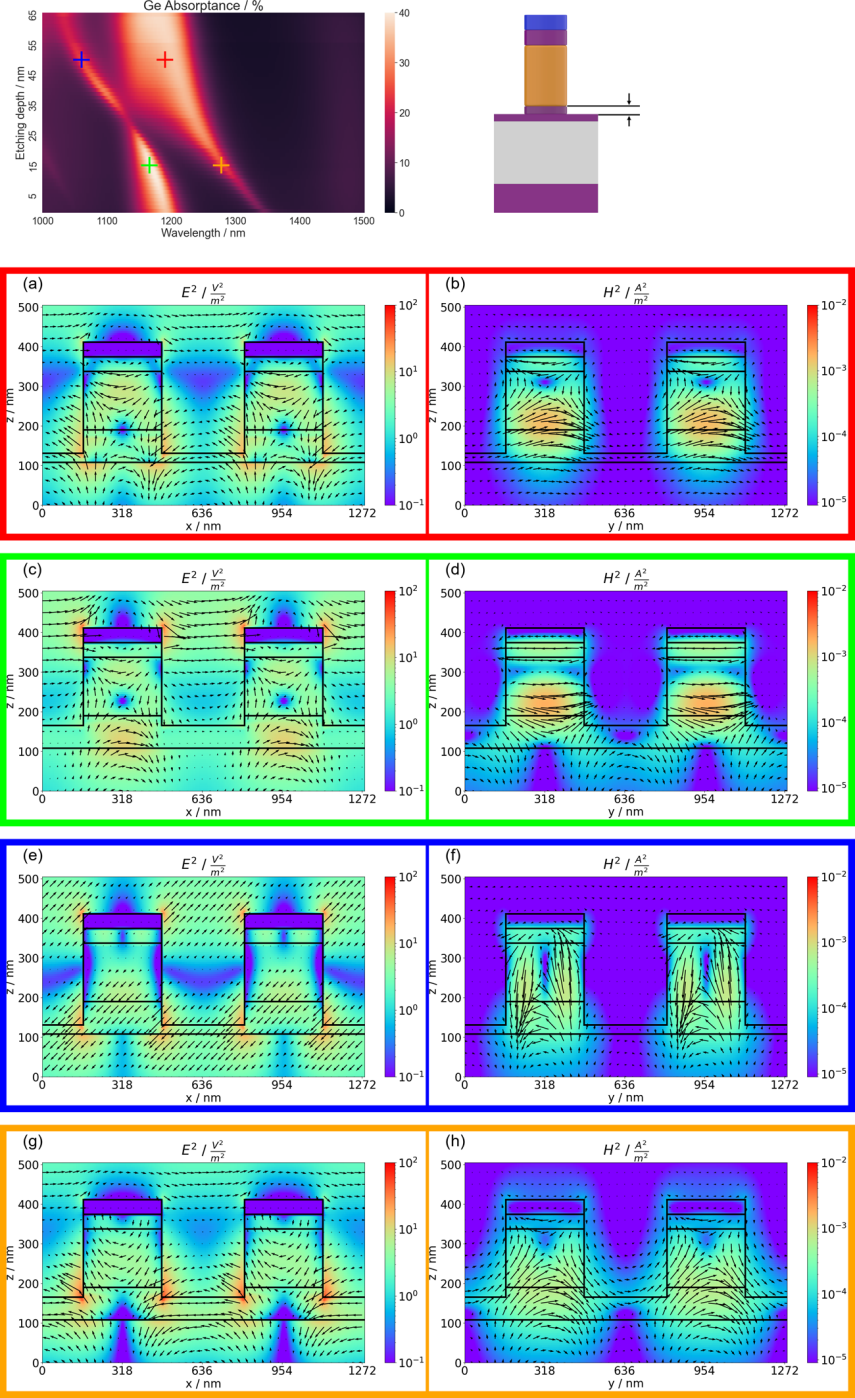
Electric (magnetic) fields in a *y*-(*x*)normal cross section. While the fields obtained at a wavelength of 1190 nm and etching depth of 50 nm ((a) and (b); red border) as well as at a wavelength of 1166 nm and etching depth of 15 nm ((c) and (d); green border) show characteristics of an electric quadrupole resonance, the fields obtained at a wavelength of 1060 nm and etching depth of 50 nm ((e) and (f); blue border) as well as at a wavelength of 1278 nm and etching depth of 15 nm ((g) and (h); orange border) show characteristics of a magnetic quadrupole resonance.

Our analysis indicates that the optical behavior of our hybrid metasurface derives from strong optical coupling of electric and magnetic quadrupole collective lattice resonances (EQ-CLR, MQ-CLR). Two structural aspects of our metasurface, in particular, influence its optical properties: on the one hand, the presence of the Al nanoparticle array induces interactions between the EQ-CLR and MQ-CLR and, on the other hand, adjusting the continuous Si layer below the Ge strongly affects their spectral separation.

The presence of the Al nanoparticle array has a pronounced influence, in particular, on the EQ-CLR, and strong electric field enhancement can be observed in the vicinity of the Al nanoparticle at the resonance wavelength (Figure 3c). The multipole analysis reveals that the spectral positions of the EQ-CLR resonances in the Ge disks and of the ED-CLR in the Al disks coincide (Figure 2d). Indeed, our FDTD simulation results show that the Al nanoparticle array induces a strong spectral shift in the EQ-CLR (from 1310 to 1190 nm) when comparing the heat maps of the hybrid and the all-dielectric metasurface (Figure 2b,c), indicating a possible hybridization of ED-CLR in the Al disks and EQ-CLR in the Ge disks in the hybrid metasurface. Furthermore, in the hybrid metasurface, the width of the EQ-CLR resonance is strongly reduced compared to the all-dielectric metasurface (Figure S6 in the Supporting Information). The coupling between Al nanoparticles and the dielectric structure leads to coupling between the EQ-CLR and the MQ-CLR, visible as avoided resonance crossing in the spectra (Figure 2b), while these resonances do not interact in the all-dielectric metasurface.

Field confinement in Ge is affected not only by the presence of the Si spacer layer between the Al disk and the Ge nanocylinder, but also by the continuous Si layer below the Ge nanocylinders. This can lead to an impact of the etching depth on optical properties of the metasurface.^22^ Indeed, the spectral position of the MQ-CLR is strongly influenced by the etching depth: The presence of a continuous Si layer underneath the Ge disks mediates coupling between the disks as indicated by the presence of a nonvanishing electric field amplitude within the bottom Si layer at resonance (Figure 3). Varying the etching depth can be considered to modify the dielectric environment of the Al/Ge nanoresonators, which can induce a shift in the spectral position of the lattice resonance. This shift is more pronounced for the MQ-CLR than for the EQ-CLR as a consequence of the different position-dependent electromagnetic field distributions within the structure. As a result, a variation in the etching depth strongly affects the spectral distance between the EQ-CLR and the MQ-CLR.

A comparison between measured absorptance spectra for the fabricated hybrid metasurface and simulated spectra at vertical incidence (Figure 4a) shows good agreement between peak positions at ∼1115 nm. Here, a pillar diameter of 270 nm and an etching depth of 65 nm assumed in the simulation yielded the best agreement with measured results. However, the measured absorptance peak is broadened compared to that in simulation. This can be attributed to variations in the fabrication process, most notably a variation in the diameters of the individual pillars of the metasurface. Furthermore, as previous device fabrication processes have shown, the dry etching step leads to the appearance of both a slight underetching of the Ge layers compared to the Si cap as well as a pedestal at the pillar bottom.^30,31^ Compared to the simulated absorptance, the measured absorptance is increased for all wavelengths. This can be attributed to scattering losses; those are not accounted for in our de-embedding approach, which extracts the absorptance from measured reflectance and transmittance spectra. Nonetheless, both experiment and simulation show a pronounced absorptance peak; this is relevant for possible applications in on-chip spectroscopy and, notably, can be obtained from a structure with a Ge thickness of only 160 nm. This makes our approach potentially more suitable for on-chip integration than concepts relying on wavelength-selective absorption in Ge vertical nanowires with typical heights between 1 and 2 μm.^31,32^

**Figure 4:**
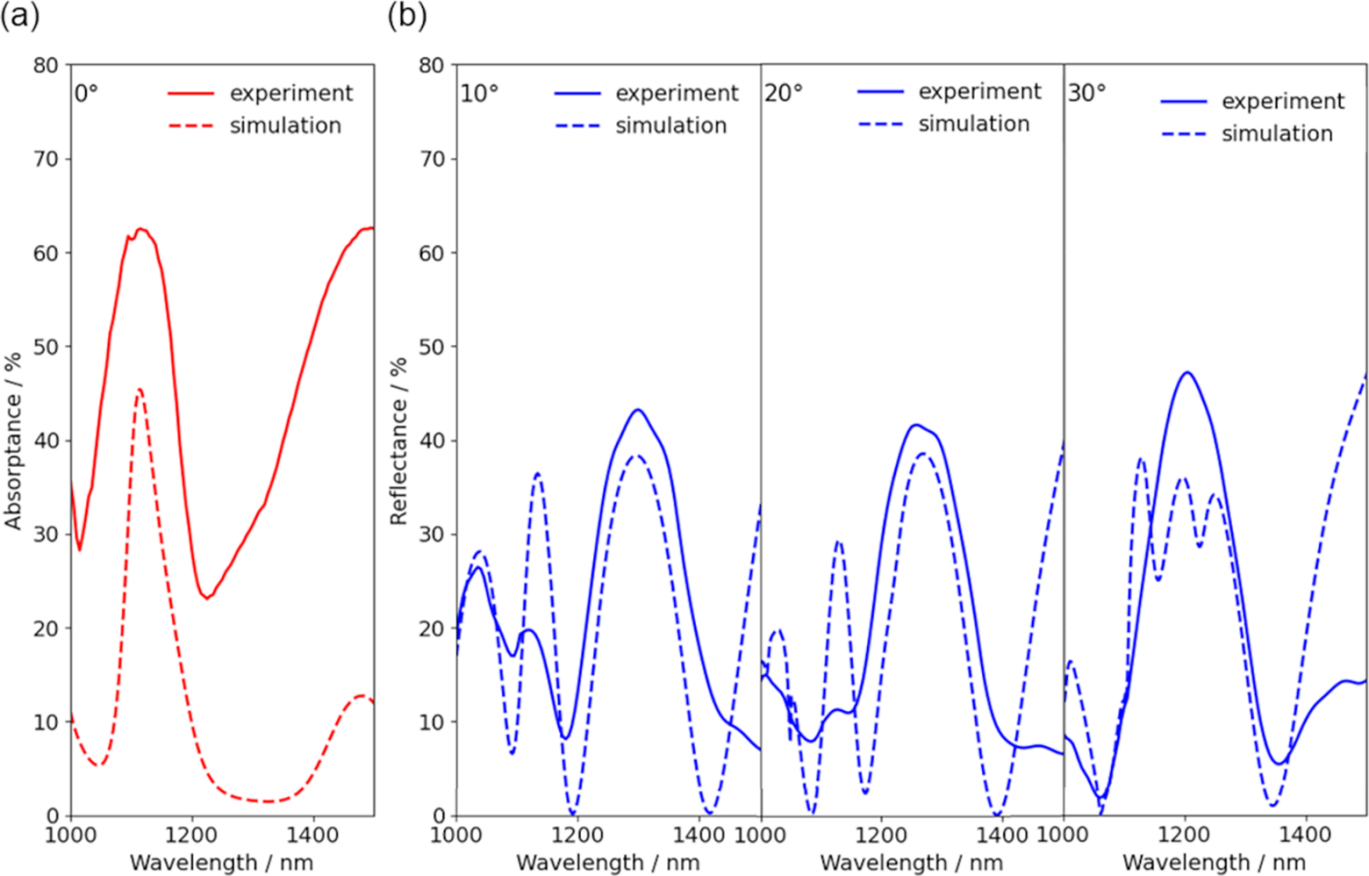
(a) A comparison of measured (solid line) and simulated (dashed line) absorptance spectra shows good agreement of peak positions at ∼1115 nm, while the peak width is noticeably larger in the measured absorptance as a consequence of fabrication imperfections. (b) A comparison of measured (solid lines) and simulated (dashed lines) reflectance spectra under different angles of incidence and using s-polarized light shows good agreement in the positions of the main peaks and dips.

To further investigate the optical properties of the fabricated metasurface, reflectance measurements under different angles of incidence with s-polarized light were also compared to simulation results (Figure 4b). Again, there is good agreement between the spectral positions of the main peaks and dips for the measured and simulated reflectance spectra, but as a consequence of fabrication imperfections in the fabricated metasurface, not all peaks are clearly visible in the measured spectra compared to simulation. Results for p-polarized light are given in the Supporting Information (Figure S7).

Device integration typically requires the use of continuous contacting layers that connect all individual elements of the metasurface, however, the presence of these continuous layers can be detrimental to the optical properties of metasurfaces by making those highly susceptible to variations in the fabrication process.^22^ In our structures, the dependence of the optical properties of the metasurface on the structure of a continuous connecting layer can be exploited for tunable metasurfaces. As our simulations show, avoided resonance crossing can be induced by replacing the bottom Si layer with a material whose permittivity can be adjusted (Figure 5). The selected permittivity range is large; however, transparent conducting oxides such as ITO can exhibit refractive index changes above unity. For example, a change of the refractive index between 0.5 and 1.95 at a wavelength of 1310 nm has been predicted for ITO if the carrier concentration is varied between 10^19^ and 10^21^.^33^ In our hybrid metasurface, tuning across a level crossing can, in principle, be achieved by varying the refractive index of a continuous layer; this opens up the possibility of electric tuning of the metasurface properties via a back-gate. Systems in which resonances of different types are strongly coupled and can be tuned have also been proposed for tunable phase modulation.^34^ In this context, it would be interesting to investigate whether strong phase modulations in our hybrid metasurface upon tuning across the level crossing can also be induced and exploited.

**Figure 5:**
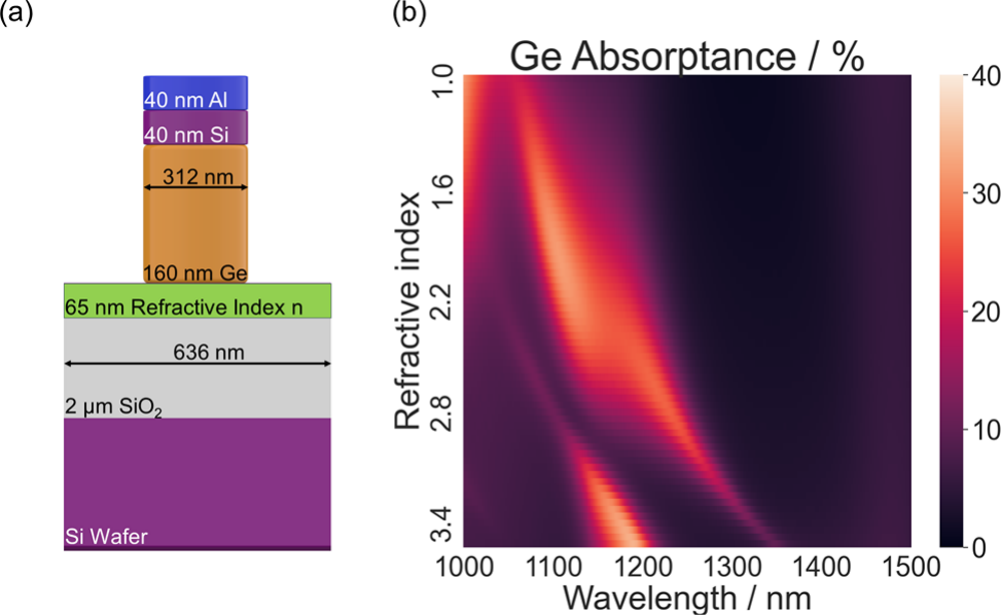
(a) Schematic cross-section of a metasurface layer stack, in which the bottom Si layer is replaced by a layer with a variable refractive index n. (b) Simulated heat maps of the absorptance in the Ge show that a variation in refractive index n leads to signatures of avoided crossing.

To conclude, we demonstrated that high absorptance peaks can be obtained in a hybrid Al/Si/Ge metal–dielectric metasurface fabricated via a straightforward top-down approach with one single etching step. The introduction of both a thin, connecting Si layer below the Ge nanocylinders and an array of Al nanodisks on top of the semiconductor nanocylinders allows us to separately adjust the spectral separation and the interactions between the electric quadrupole and magnetic quadrupole collective lattice resonances in the nanocylinder arrays. This leads to the formation of strongly coupled electric quadrupole and magnetic quadrupole lattice resonances in the wavelength range investigated (1000–1500 nm), whose spectral positions can be tuned by varying selected geometry parameters. Varying the etching depth, in particular, leads to avoided crossing of the resonances in the absorptance spectra as a result of strong optical coupling.

Our metasurface can easily be designed to exhibit absorptance peaks that show weak shifts upon variation of the etching depth; this is the geometry parameter that is most difficult to control in fabrication. This makes our hybrid metasurface more robust with respect to fabrication tolerances than its all-dielectric counterpart, in which the Al is omitted and leads to pronounced absorptance peaks and reflection dips in measured spectra of our fabricated hybrid metasurface despite fabrication imperfections.

The continuous Si layer connecting the elements of the hybrid metasurface ensures that the metasurface can be easily incorporated into optoelectronic devices. Several possibilities for applications can be envisioned. A straightforward application of our metal–dielectric metasurface to wavelength-selective photodetection can make use of a parameter regime in which absorptance peaks are weakly affected by crucial fabrication tolerances, e.g., in the etching depth. A wavelength range of 1000–1500 nm could allow us to target applications such as optical detection of water stress in plants^35^ or imaging-based plastic classification and recycling.^36^ Future investigations could also focus on applications in refractive index sensing. The presence of a continuous connecting layer, moreover, can be explored for active metasurface design if the refractive index of this layer can be varied via external gating.

The investigation of approaches to metasurface design that ensure the confinement of strong electromagnetic fields to the nanostructured elements of the metasurface even in the case when the metasurface is incorporated into device layers remains of high relevance for the realization of metasurface-based devices. Our hybrid metal–dielectric metasurface design thus constitutes an important step in making use of the cost-effective Si-based CMOS platform for versatile, metasurface-based device applications ranging from on-chip spectrometers to sensing.

## Supporting Information Available

Complex refractive indices of Al, Si, and Ge (Figure S1); Extraction of normal mode splitting from fits to hybrid modes (Figure S2); Field distributions for the all-dielectric metasurfaces (Figure S3); Heat maps of absorptance as a function of changes in geometry parameters (Figures S4 and S5); Comparison of absorptance spectra between hybrid and dielectric metasurface (Figure S6); Comparison of measured and simulated reflectance spectra for p-polarized light (Figure S7) (PDF)
