# Supplementary material for: Strong Optical Coupling of Lattice Resonances in a Top-down Fabricated Hybrid Metal–Dielectric Al/Si/Ge Metasurface
Source: Nano Lett. 2024 Mar 1;24(10):3142–9. doi: 10.1021/acs.nanolett.3c05050 (PMC10941247; doi:10.1021/acs.nanolett.3c05050)
Supplement: Supplementary file 1 — nl3c05050_si_001.pdf [file nl3c05050_si_001.pdf]

Supporting Information to

Strong optical coupling of lattice resonances in a top-down fabricated hybrid metal-dielectric Al/Si/Ge metasurface

Paul Oleynik[1], Fritz Berkmann[2], Sebastian Reiter[1], Jon Schlipf[1], Markus Ratzke[1], Yuji Yamamoto[3], Inga Anita Fischer[1]

[1]*Experimentalphysik und Funktionale Materialien, Brandenburgische Technische Universität Cottbus-Senftenberg, Erich-Weinert-Straße 1, 03046, Cottbus, Germany*

[2]*Department of Physics, Sapienza University of Rome, 00185 Rome, Italy*

[3]*IHP–Leibniz Institut für Innovative Mikroelektronik, Im Technologiepark 25, 15236, Frankfurt (Oder), Germany*

1. Refractive indices of Al, Si and Ge

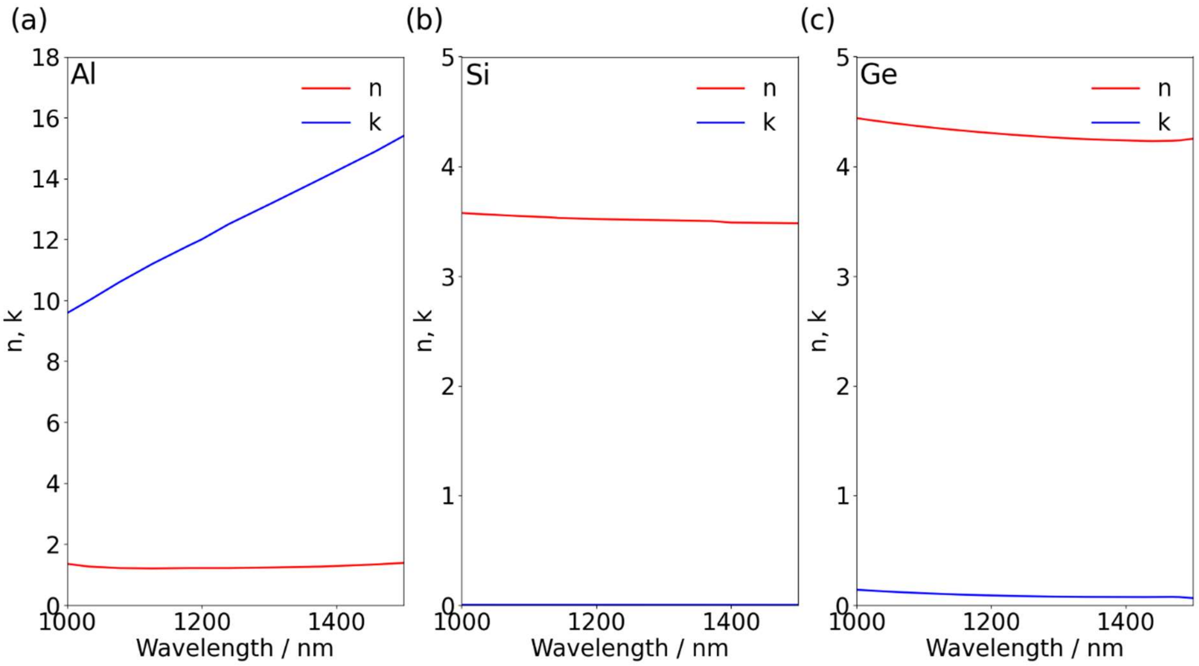

Figure S1: Complex refractive indices of (a) Al, (b) Si and (c) Ge as used in FDTD simulations.

2. Normal mode splitting

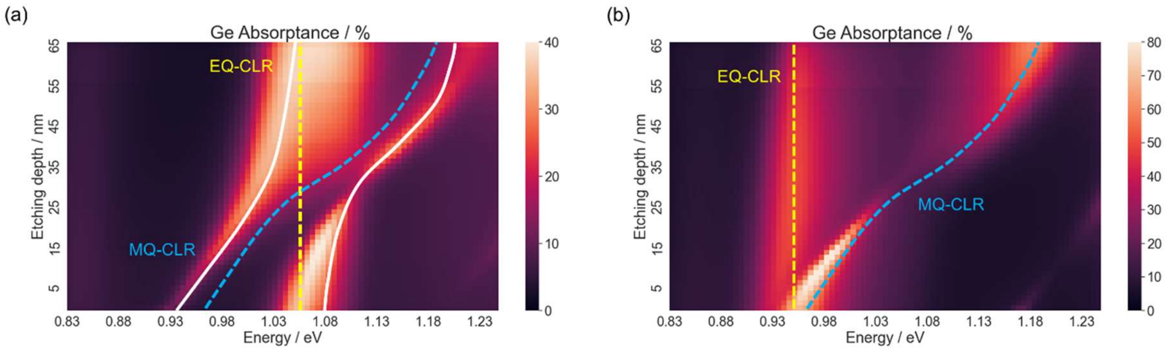

Figure S2: Absorptance in the Ge of the (a) hybrid and (b) the dielectric metasurface (without Al cap) as a function of photon energy and etching depth. Solid lines indicate fit results to the coupled resonances, while dashed lines indicate the positions of the uncoupled resonances.

In the coupled, lossless oscillator model, the energies $E_{\pm}$ of the coupled modes (solid white lines in Fig. S2 (a)) are given by

$$E_{\pm} = \frac{E_{EQ-CLR} + E_{MQ-CLR}}{2} \pm \sqrt{\frac{\left(E_{EQ-CLR} - E_{MQ-CLR}\right)^2}{4} + g^2}, \quad (1)$$

where E_{EQ-C} (d) and E_{MQ-C} (d) are the energies of the uncoupled collective lattice modes as a function of etching depth d (Fig. S2 (b)) and the coupling strength g is a fit parameter that is adjusted so that the energies $E_{\pm}$ provide a good fit to the upper and lower branches of the resonance energies of the coupled system. In our case, while the dependence of E_{EQ-CL} on the etching depth is negligible, E_{MQ-} (d) shows a non-linear dependence on d that we fit as follows:

$$E_{MQ-CLR}(d) = d^3 \cdot 1.5489 \cdot 10^{-6} \frac{eV}{nm^3} + d^2 \cdot 1.5773 \cdot 10^{-4} \frac{eV}{nm^2} - d \cdot 5.2475 \cdot 10^{-4} \frac{eV}{nm} + 0.95889\, eV \quad (2)$$

The energies $E_{EQ-CLR}(d)$ and E_{MQ-CL} (d) are shown in Fig. S2 (b) as dashed lines. The solid lines in Fig. S2 (a) show results for $E_{\pm}$ according to Eq. (1) and with g = 40 meV. As a result, we obtain a normal mode splitting ΔE = $2g$ = 80 meV for our system.

3. Field distributions for the all-dielectric metasurface

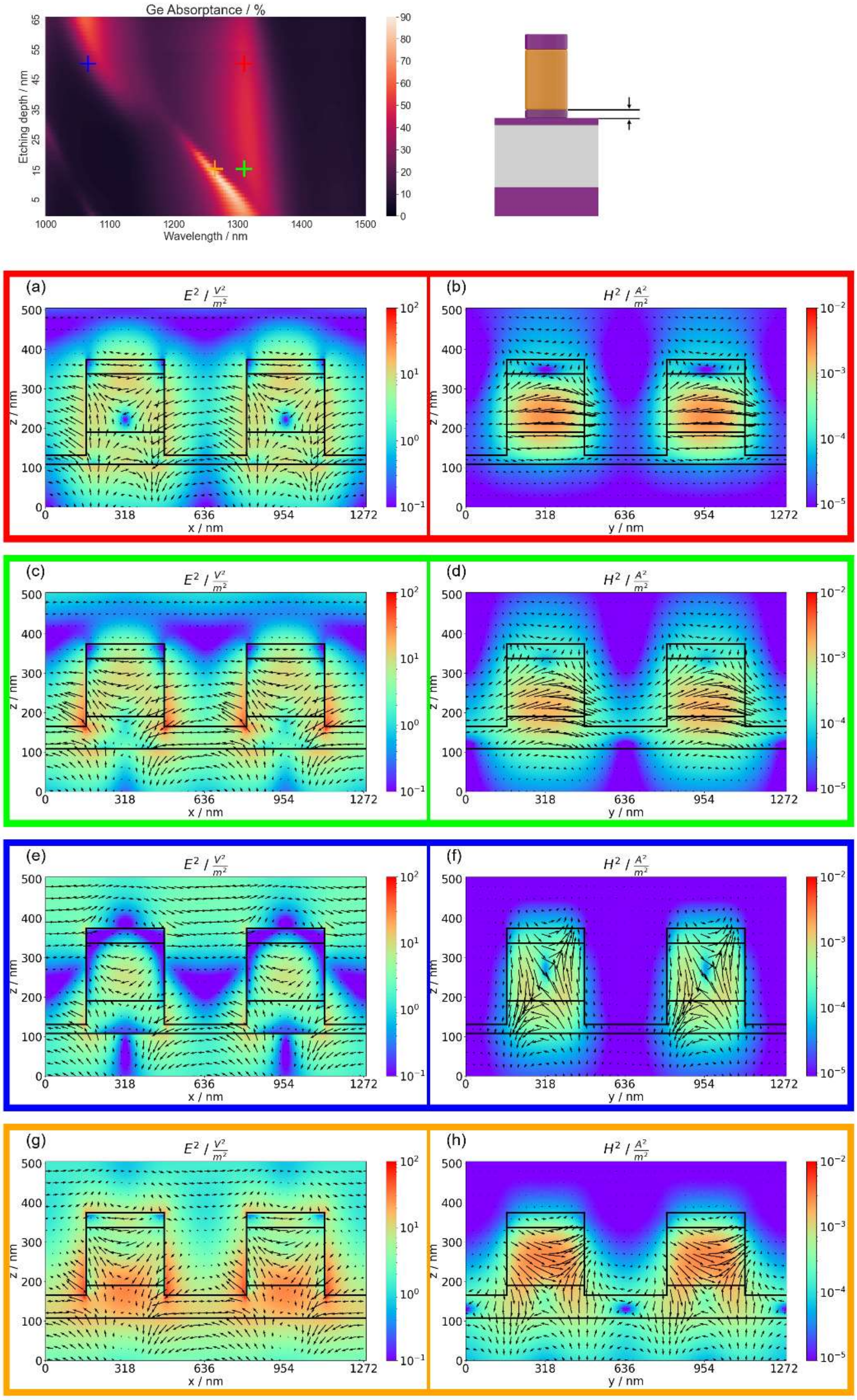

Figure S3: Electric (magnetic) fields in a y-(x-)normal cross section of the dielectric metasurface, i.e. where the top Al disk has been omitted. The fields obtained at a wavelength of 1310 nm and etching depth of 50 nm ((a) and (b); red border) as well as at a wavelength of 1310 nm and etching depth of 15 nm ((c) and (d); green border) show characteristics of an electric quadrupole resonance. The fields obtained at a wavelength of 1065 nm and etching depth of 50 nm ((e) and (f); blue border) as well as at a wavelength of 1264 nm and etching depth of 15 nm ((g) and (h); orange border) show characteristics of a magnetic quadrupole resonance.

Heat maps of absorptance as function of changes in geometry parameters

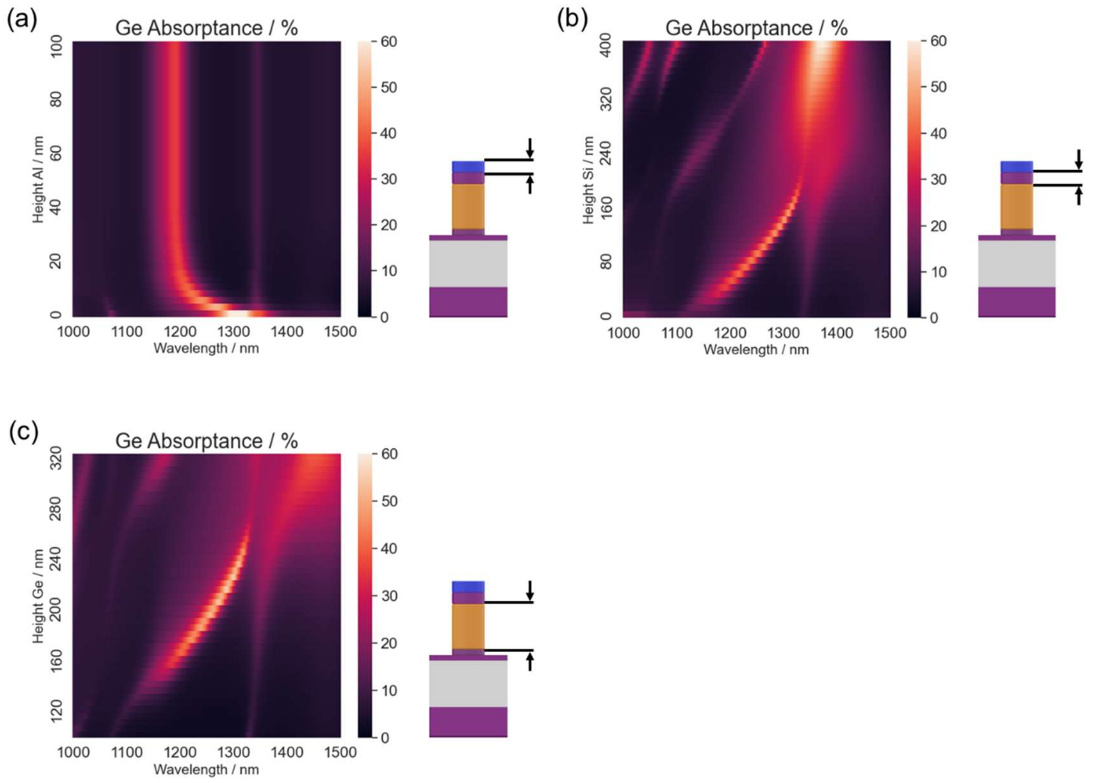

Figure S4: Simulated absorptance spectra as a function of changes in thicknesses of (a) the top Si disk, (b) the Ge disk and (c) the Al disk. Both changes in the thickness of the top Si disk and of the Ge disk lead to avoided crossing of resonances, while the Al thickness leaves the positions of the absorptance peaks unchanged once it exceeds 10 nm.

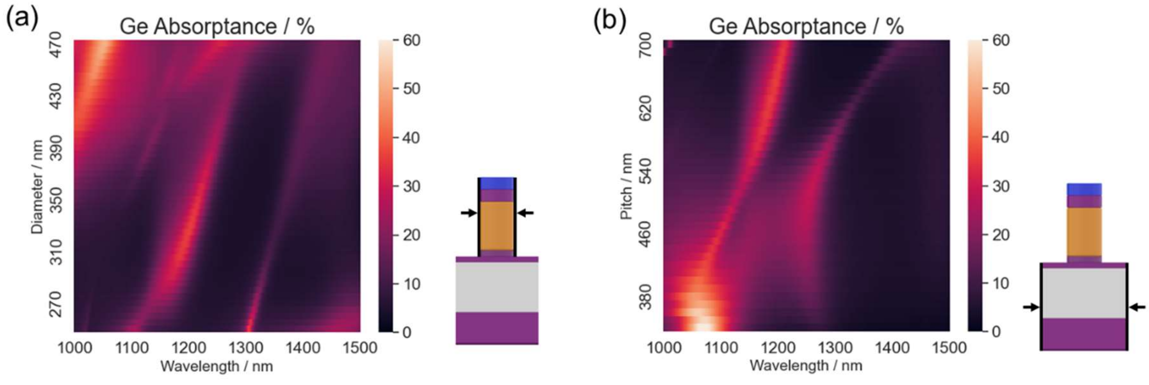

Figure S5: Simulated absorptance spectra as a function of changes in the lateral geometry parameters (a) disk diameter and (b) lattice pitch. While a change in lattice pitch also induces avoided crossing of resonances, a change in disk diameter shifts the absorption peaks but leaves their spectral separation largely unchanged.

4. Comparison of absorptance spectra between hybrid and dielectric metasurface

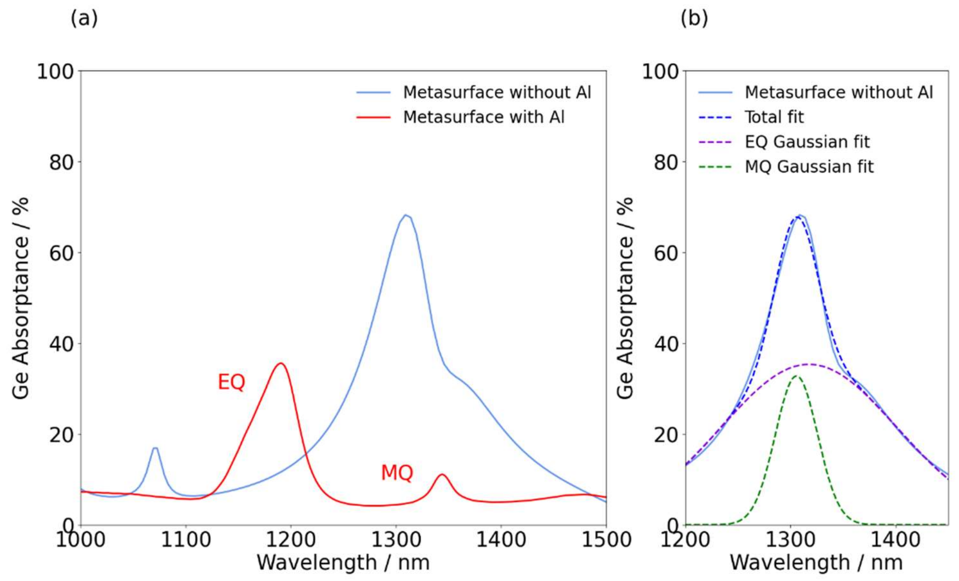

Figure S6: (a) A comparison of the absorptance spectra shows pronounced changes in peak height and width for the hybrid metasurface (solid line) as compared to the dielectric metasurface (dashed line). A fit to the absorptance for the dielectric metasurface without the Al using two Gaussian peaks for the overlapping EQ and MQ contributions is shown in (b). All spectra were obtained for an etching depth of 0 nm into the bottom Si layer.

5. Comparison of measured and simulated reflectance spectra for p-polarized light

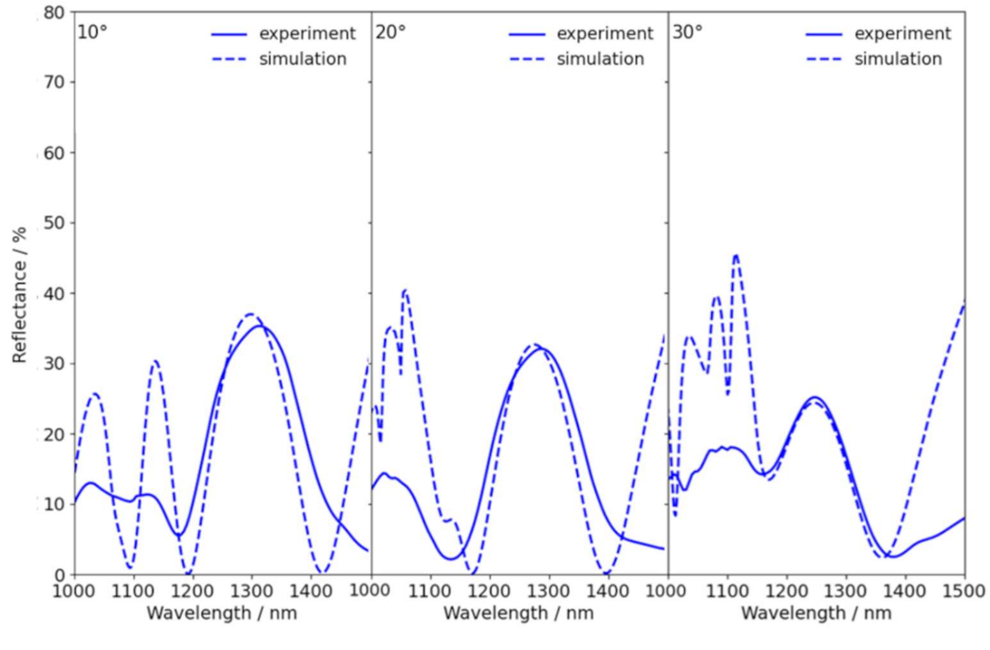

Figure S7: A comparison of measured (solid lines) and simulated (dashed lines) reflectance spectra under different angles of incidence and using p-polarized light shows good agreement in the positions of the main peaks and dips, while the narrow peaks in a wavelength range of 1000 – 1150 nm are not well seen in the measured spectra as a result of fabrication inhomogeneities.
